# Reframing disaster simulation as a translational systems intervention: a design-based comparative analysis of MRMI and the Emergo Train System (ETS)

**DOI:** 10.1186/s12873-026-01699-1

**Published:** 2026-07-22

**Authors:** Khorram-Manesh Amir, Carlström Eric

**Affiliations:** 1 Institute of Clinical Sciences, Sahlgrenska Academy, University of Gothenburg, Gothenburg, Sweden; 2 Center for Disaster Medicine, University of Gothenburg, Gothenburg, Sweden; 3 Gothenburg Emergency Medicine Research Group, Sahlgrenska University Hospital, Gothenburg, Sweden; 4 Institute of Health and Care Sciences, University of Gothenburg, Gothenburg, Sweden

**Keywords:** Design-based research, Disaster simulation, Disaster preparedness, Emergency management, Resilience engineering, Socio-technical systems, Translational simulation

## Abstract

**Background:**

Disaster simulation is central to emergency preparedness; however, many simulation designs insufficiently represent disasters as prolonged, system-disruptive, and ethically complex events. Exercises frequently emphasize mass-casualty throughput, rely on simplified assumptions, and evaluate success primarily in terms of task completion rather than system performance and organizational learning. This study examines how disaster simulation design shapes preparedness outcomes and explores how simulation may be reframed as a translational systems intervention.

**Methods:**

A qualitative design-based research approach was used to conduct an inductive comparative framework analysis of two established disaster simulation paradigms: Medical Response to Major Incident (MRMI) and the Emergo Train System (ETS). Documentary sources included curricula, instructional materials, scenario templates, published evaluations, disaster evidence literature, and World Health Organization Emergency Medical Team standards. Simulation design features were extracted and compared using constant comparative analysis, and the findings were subsequently interpreted through socio-technical systems and resilience engineering perspectives.

**Results:**

Four recurrent design-level tensions were identified using harmonized terminology: (1) divergent disaster conceptualization (governable surge versus unstable system disruption); (2) fidelity emphasis imbalance (cognitive versus behavioral realism); (3) incident-centric temporal framing; and (4) inconsistent translation into organizational learning. Published evaluation reports suggest that MRMI may support governance alignment and coordinated surge reasoning, whereas ETS may provide greater operational realism and more opportunities to expose teamwork under stress. Neither paradigm alone appears to fully address all design requirements relevant to sustained disaster-level system resilience.

**Conclusions:**

MRMI and ETS appear complementary but individually incomplete. A staged hybrid architecture integrating governance modeling, operational stress testing, longitudinal degradation modeling, and structured reflective translation is proposed as a design-oriented framework. This framework may help position disaster simulation as a translational systems intervention that connects exercise performance to potentially measurable organizational preparedness and system resilience, while still requiring empirical validation in practical settings.

**Supplementary Information:**

The online version contains supplementary material available at 10.1186/s12873-026-01699-1.

## Background

Disasters and emergencies impose complex and resource-intensive demands on healthcare and emergency response systems [1, 2]. Unlike routine emergencies, disasters involve uncertainty, resource scarcity, ethical complexity, and disruption of organizational and societal functions. These conditions challenge not only clinical care but also leadership, coordination, governance, and public trust [3, 4]. Disaster response is therefore not merely a clinical issue but also a multi-layered operational and public health challenge in which interagency coordination, logistics, communication systems, and workforce sustainability shape outcomes and overall system stability [3].

Most clinicians and responders, however, are trained in stable environments characterized by predictable staffing and intact infrastructure. This creates a persistent mismatch between training contexts and disaster realities—particularly when disasters unfold over time through cascading failures and competing operational priorities. In such contexts, preparedness depends less on isolated technical competence and more on system performance: the ability of organizations to adapt as resources degrade, information becomes fragmented, and ethical pressures intensify [2, 5, 6].

Because real disasters are rare and ethically unsuitable for experiential training, simulation has become an essential surrogate for preparedness [5, 6]. Simulation enables the rehearsal of decision-making, coordination, and leadership within controlled conditions where complex dilemmas can be explored without causing harm [7–9]. However, the effectiveness of simulation depends heavily on design. Many exercises emphasize mass-casualty throughput and task completion, thereby reproducing multiple-casualty incidents rather than prolonged, system-disruptive disasters. Such exercises often rely on hypothetical casualty profiles and idealized resource assumptions rather than empirically grounded disaster evidence [10–12]. This limitation is important because disasters primarily stress systems rather than individuals, exposing coordination breakdowns, resource constraints, and competing priorities that extend beyond protocol-based instruction [13]. Disaster response also entails cognitive and ethical challenges—such as decision-making under uncertainty and prioritization under scarcity—which require structured experiential engagement and critical reflection [7, 14].

Two recurring limitations constrain disaster simulation as a preparedness strategy. First, many simulation designs assume stable infrastructure, adequate staffing, and predictable command structures, despite evidence that real disasters involve nonlinear casualty flows, fragmented information, workforce attrition, supply chain disruption, and evolving operational constraints [15–17]. Second, even high-fidelity exercises often focus primarily on the initial impact phase, neglecting fatigue, ethical escalation, secondary events, and recovery dynamics. Simulations that conclude once immediate surge pressures subside fail to represent longitudinal system degradation and broader resilience challenges [5, 9, 18]. Together, these limitations contribute to a translation gap: simulations may generate meaningful learning experiences without consistently producing actionable organizational improvements, such as revisions to disaster plans, logistical structures, governance pathways, or quality improvement processes. In this study, “translational” refers to the explicit linkage between simulation activities and system-level change. This means that insights generated during simulation are converted into measurable modifications in organizational structures, operational processes, and preparedness capacity, rather than remaining solely at the level of individual learning.

To examine how simulation design shapes preparedness, this study analyzes two influential disaster simulation paradigms developed by Professor Sten Lennquist and collaborators during different periods: Medical Response to Major Incident (MRMI) and the Emergo Train System (ETS) [19–22]. Although the two paradigms differ in format and emphasis, both represent established interprofessional simulation approaches designed to support major incident and disaster preparedness. Both occupy a broadly intermediate level of simulation fidelity, simplifying some elements of real-world disaster response while preserving selected features of coordination, decision-making, resource management, and patient-flow complexity. These similarities make the paradigms analytically comparable while their different design priorities allow comparison of complementary dimensions of disaster response.

MRMI is a multi-agency training program designed to standardize major incident management through a “whole chain of response” approach spanning scene management, patient transport, and hospital care. Using the MACSIM system with validated patient cards and structured role-based coordination, MRMI emphasizes triage logic, command structures, and governance under surge conditions [7, 17, 19, 22–26]. Its principal strength lies in normative system modeling—clarifying doctrine and roles. However, exposure to operational friction may vary depending on how the simulation is implemented.

ETS, developed earlier, is a tabletop simulation methodology, emphasizing operational realism at lower logistical costs [20, 21]. Using whiteboards and magnetic symbols representing patients, staff, and resources, ETS highlights patient flow, bottlenecks, time pressure, and coordination breakdowns across functional units [20, 21, 27, 28]. The system functions as a form of experiential stress testing by making visible how work is performed under pressure and how system performance degrades when resources are constrained [27, 29–31]. However, governance structures and ethical escalation pathways are often modeled less explicitly. This may limit the translation of experiential insights into structured organizational change.

**Fig. 1:**
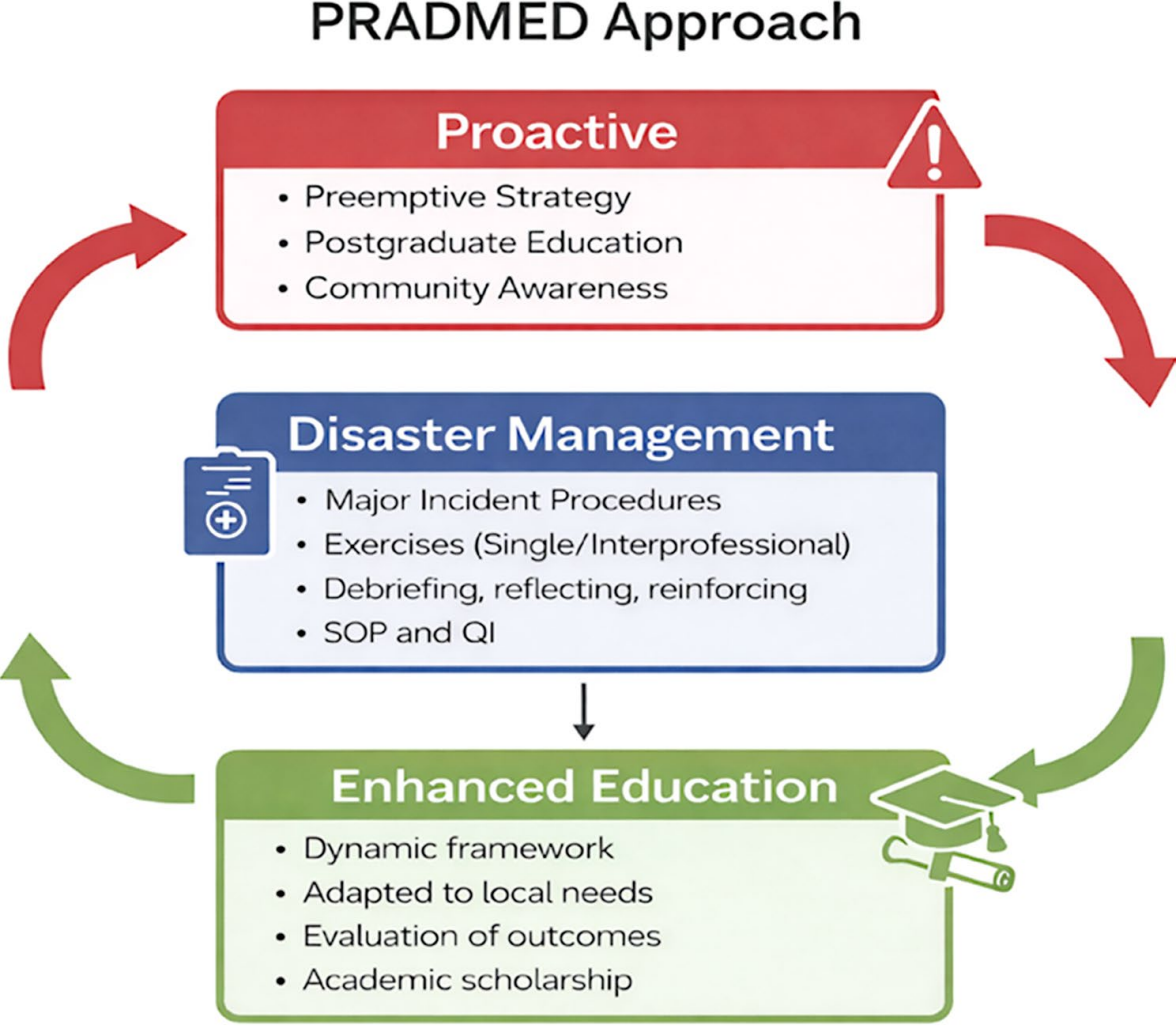
The PRAD-MED approach

Recent modular disaster education programs demonstrate the feasibility of staged training models that integrate doctrine and structured reflection. For example, PRAD-MED (PReparedness And Disaster MEDicine) combines formal disaster medicine instruction with MRMI-based simulation and reflective components, including Three-Level Collaboration (3LC) cycles that support multidisciplinary coordination and iterative organizational learning [7, 32]. Figure 1 provides a simplified visual abstraction of the broader PRAD-MED model rather than a complete 5-staged operational flowchart.

The PRAD-MED, a proactive postgraduate education in disaster medicine and preparedness for enhanced disaster management, approach is described as a staged, longitudinal, and transdisciplinary educational model designed to develop disaster medicine competence and strengthen organizational preparedness. The model comprises five sequential but iterative stages: (1) prepare and anticipate, focusing on foundational knowledge, risk awareness, and system-level preparedness; (2) rehearse and respond, emphasizing simulation-based and interprofessional experiential learning; (3) analyze and reflect, incorporating structured debriefing, feedback, and critical reflection; (4) develop and integrate, translating learning into organizational improvements such as quality improvement initiatives, protocol revisions, and policy integration; and (5) measure and sustain, evaluating outcomes, monitoring performance, and supporting continuous learning and capability maintenance.

Key features of the model include its proactive and longitudinal structure, transdisciplinary integration across clinical, operational, and public health domains, and its emphasis on linking experiential learning to organizational systems, including Standard Operating Procedures (SOPs) and Quality Improvement (QI) processes. The visual elements summarize the model’s core educational logic: needs assessment and preparation; simulation-based, interprofessional learning; structured reflection; and translation into SOP/QI processes that support sustained preparedness. The intended outcome is the development of a competent, collaborative, and adaptive disaster workforce capable of supporting system-level preparedness and enhancing resilience.

3LC emphasizes reflection across operational, tactical, and strategic levels by incorporating structured pauses during exercises to reassess decisions and communication pathways [33]. Such pauses may support deliberate reflection and shared sense-making. However, they may also reduce operational realism by providing opportunities for recovery and reassessment that would be limited during real incidents. This design trade-off may be educationally valuable for learning and coordination, but it may be less effective for cultivating uninterrupted stress adaptation and resilience under sustained operational pressure. Although such approaches strengthen governance-oriented alignment and reflective learning, they rarely incorporate ETS-style operational stress testing as a distinct simulation design component.

This creates an opportunity for a staged hybrid architecture that integrates normative system modeling, experiential stress testing, longitudinal degradation modeling, and structured reflective translation. Accordingly, this study conducts a qualitative, design-based comparative analysis of MRMI and ETS to clarify how simulation design choices shape preparedness outcomes. It aims to (1) identify recurring structural tensions related to divergent disaster conceptualization, fidelity emphasis imbalance, incident-centric temporal framing, and inconsistent translation into organizational learning and (2) develop a staged hybrid simulation framework that strengthens disaster-level system preparedness, ethical decision-making, and organizational learning. The proposed framework aligns with (World Health Organization) WHO Emergency Medical Team (EMT) standards [34] and positions disaster simulation as a socio-technical resilience intervention in which safety and effectiveness emerge from the interaction of people, technology, and governance under pressure [35–38].

## Methods

### Study design

This study employed a design-based research (DBR) framework to analyze and refine disaster simulation design as a system-level preparedness intervention [39, 40]. DBR was selected because it supports the theory-informed examination of real-world educational interventions and the development of actionable design guidance. Within this framework, the study adopted an inductive constant comparative analytic strategy to examine and compare the architectural features of two established disaster simulation paradigms: Medical Response to Major Incident (MRMI) and the Emergo Train System (ETS).

MRMI and ETS were selected as information-rich cases because they represent influential, operationalized disaster simulation paradigms with clearly articulated doctrines, established training formats, and international adoption [17–31]. They also embody contrasting design logics—MRMI emphasizes normative governance and chain-of-response modeling, and ETS emphasizes operational friction, flow constraints, and behavioral performance under time pressure. These contrasting characteristics make the paradigms analytically well-suited for identifying structural tensions at the design-level.

The first author led the analysis in a theorist–designer role, systematically mapping disaster-response requirements onto simulation architectures and iteratively moving between empirical evidence, operational benchmarks, and design interpretations. The second author independently reviewed extraction matrices and analytic memos, contributing to category refinement and challenging interpretive assumptions through structured analytic dialogue. The primary outputs of the DBR process were: (1) a comparative analytic framework for examining disaster simulation design and (2) a staged hybrid simulation architecture intended to strengthen translational learning and organizational preparedness [41].

### Unit of analysis

The unit of analysis comprised discrete simulation design features that influence preparedness outcomes [42, 43]. These features were defined a priori and used as extraction and coding categories:

1. **Governance and command structure** (roles, escalation pathways, accountability logic, interagency interfaces).
2. **Scenario architecture** (modules/locations represented, task sequencing, inject structure, and decision points).
3. **Patient modeling and fidelity** orientation (representation of patient trajectories, physiological change processes, triage rules, documentation systems, simulation modalities, and fidelity orientation (e.g., tabletop, card-based, role-based, or mixed formats, and the degree to which physical, conceptual, psychological, functional, or sociological realism was emphasized).
4. **Resource and capacity modeling** (staffing, space, equipment, transport, consumables, surge capacity, and bottleneck representation).
5. **Stress-induction mechanisms** (time compression, information distortion, competing priorities, noise/overload proxies, and disruption injects).
6. **Temporal scope** (incident window vs. prolonged operations; fatigue/attrition modeling; and downstream consequences).
7. **Evaluation logic and outcomes** (what counts as success/failure; individual vs. system-level metrics; and avoidable mortality vs. throughput vs. plan adherence).
8. **Debriefing and translation mechanisms** (structure, prompts, links to SOP/QI, and responsibility for follow-up actions).

### Data sources and sampling strategy

A purposive sampling strategy was used to assemble a documentary corpus that captured the formal design doctrines and underlying theories of action of each simulation paradigm. Sources were selected to represent: (i) how MRMI and ETS are specified and delivered (official and instructional materials); (ii) empirical and operational evidence describing real-world system constraints, surge dynamics, and failure mechanisms; (iii) external benchmarks for coordination, governance, and clinical management; and (iv) simulation and systems scholarship used to interpret simulation design features and their underlying mechanisms. Data sources comprised four categories:

1. **Paradigm documents**: Official MRMI and ETS materials and descriptions, supplemented by foundational publications describing the development, course structure, implementation, and application of MRMI/MACSIM and ETS [17, 19, 20, 22–30].
2. **Disaster evidence and simulation design evidence**: Empirical studies used to ground scenario assumptions in observed system constraints (e.g., surge-capacity determination and assessment), as well as evidence informing simulation features linked to practitioner learning [5, 9, 15–18, 21, 24, 26, 31–33].
3. **External standards and ethics**: The WHO Emergency Medical Team (EMT) minimum standards and disaster ethics literature used to benchmark coordination, clinical governance, and quality systems [10, 14, 34].
4. **Simulation and systems scholarship**: Literature addressing disaster simulation modalities and fidelity orientation, low- and high-fidelity approaches, and socio-technical/cognitive mechanisms—including structured reflection in team and command-and-control training. These sources were used to interpret key constructs and validate analytic categories [5–9, 11, 12, 18, 21, 29–32].

The final documentary corpus comprised 29 sources: seven MRMI-related documents, six ETS-related documents, and 16 additional benchmark/theoretical sources. The additional sources comprised three external standards/ethics sources and 13 sources addressing disaster evidence, simulation design, simulation fidelity, and socio-technical or resilience perspectives. To enhance reproducibility, a source inventory is provided in Supplementary Table S1. The inventory identifies each documentary source by title or descriptive label, source type, access method, access date, and access status (public, internal, or collaborative). Access-controlled materials are described at a level that preserves confidentiality while still clarifying their analytic contribution.

**Inclusion criteria** for paradigm documents were: (i) explicit description of scenario conduct, roles, patient modeling, or evaluation processes; and/or (ii) sufficient operational detail to extract simulation design features (e.g., timing logic, resource accounting, and decision rules).

**Exclusion criteria** included duplicative versions without substantive changes; promotional materials lacking operational detail; and documents not addressing healthcare/emergency response functions.

The documentary corpus (7 MRMI-related documents, 6 ETS-related documents, and 16 additional benchmark and theoretical sources) was assembled through a combination of institutional library access, publicly available materials from official course providers, and, where applicable, direct collaboration with relevant training organizations. Proprietary training materials were accessed through established academic and professional partnerships with institutions involved in disaster preparedness and simulation-based education. This approach was intended to ensure comprehensive coverage and reproducibility while acknowledging the constraints of access-controlled materials.

Published participant feedback and course evaluations were treated as secondary documentary evidence rather than primary human subjects’ data. These materials were used to contextualize simulation design features (e.g., perceived realism, coordination value, and confidence effects) rather than to evaluate effectiveness. The extracted secondary documentary evidence used to contextualize perceived strengths and limitations of each paradigm is presented in Supplementary Table S2.

### Data management and extraction

All included documents were reviewed systematically and extracted into a structured design extraction matrix (audit trail). For each source, relevant content was mapped to the unit-of-analysis domains above, with verbatim excerpts and page or section identifiers recorded. Analytic memos were used to document emerging interpretations, areas of uncertainty, and preliminary comparisons between the two paradigms.

**Fig. 2:**
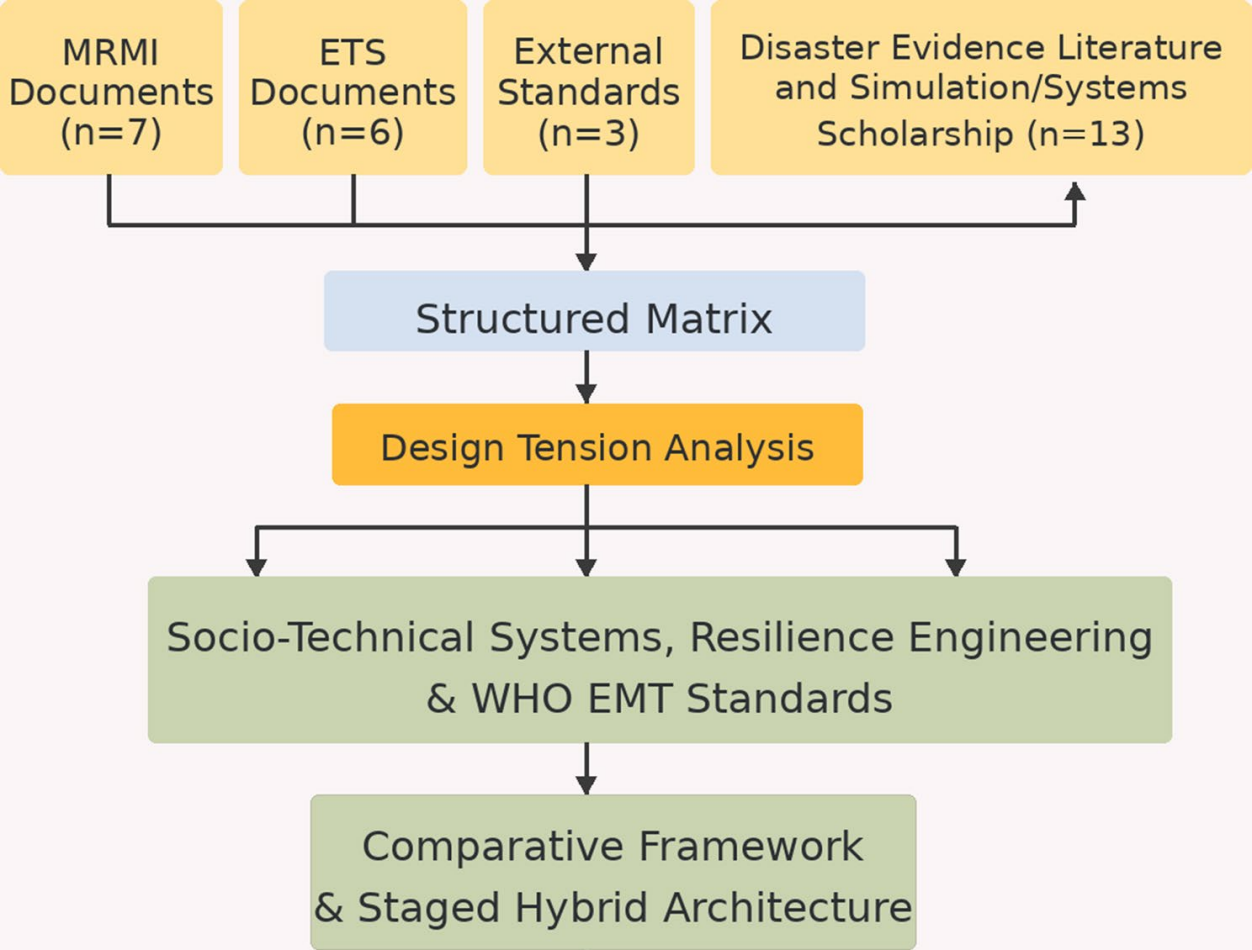
Flow chart of the design-based comparative analytic process. Documentary inputs included MRMI-related documents (*n* = 7), ETS-related documents (*n* = 6), and additional benchmark and theoretical sources (*n* = 16), comprising external standards and ethics sources (*n* = 3) and disaster evidence, exercise design, simulation, and systems scholarship (*n* = 13). Sources were extracted into a structured matrix, analyzed within and across the two paradigms, clustered into higher-order design tensions, and interpreted through socio-technical systems theory, resilience engineering, and the WHO Emergency Medical Team (WHO-EMT) standards to generate the comparative framework and staged hybrid architecture

The source inventory is presented in Supplementary Table S1, and the overall design-based comparative analytic process is summarized in Fig. 2.

## Analytic approach

An inductive constant comparative strategy was used to analyze and contrast the design architectures of MRMI and ETS [43]. The analytic process proceeded in five structured steps.

1. **Independent feature extraction: **All documents were reviewed and mapped into a structured extraction matrix (Supplementary Table S3). Design features were coded using the predefined operational domains/unit-of-analysis without imposing theoretical categories at this stage.
2. **Within-case pattern identification:** MRMI and ETS were analyzed separately to identify recurring internal design logics (e.g., definitions of success, representations of uncertainty, handling of time, and debriefing structures).
3. **Cross-case comparison:** Feature domains were compared systematically across the two paradigms to identify convergences, divergences, and trade-offs. Negative case analysis was conducted by actively searching for disconfirming evidence within the documentary corpus.
4. **Category clustering:** Recurring contrasts were iteratively grouped into higher-order design-level tensions using harmonized terminology: (i) divergent disaster conceptualization, (ii) fidelity emphasis imbalance, (iii) incident-centric temporal framing, and (iv) inconsistent translation into organizational learning. These categories emerged inductively through repeated comparison rather than being predefined.
5. **Theoretical and benchmark triangulation:** Stabilized categories were interpreted through socio-technical systems theory and resilience engineering lenses [35, 36] and benchmarked against the WHO EMT standards [34] to assess operational coherence and preparedness relevance.

### Trustworthiness, reflexivity, and traceability

Credibility and transparency were strengthened through strategies aligned with the Standards for Reporting Qualitative Research (SRQR) guidelines [43]. An audit trail was maintained through version-controlled extraction matrices, analytic memos, and iterative revisions to the framework, documenting how interpretations evolved over time.

Triangulation was conducted across documentary types (curricula/manuals, evaluation reports, disaster evidence, and standards) and across interpretive lenses [Socio-Technical Systems theory (STS)/Resilience Engineering (RE)] and operational benchmarks). Negative case analysis was used to refine emerging claims by identifying qualifying evidence and limiting overgeneralization. Benchmarking against the WHO EMT standards functioned as an external constraint on interpretation [34].

Reflexivity was addressed by acknowledging that both authors’ experience in disaster preparedness informed contextual understanding but could also introduce normative bias. To mitigate this risk, analytic claims were anchored in explicit documentary artefacts and benchmark standards. The second author’s independent review of extraction matrices and interpretive memos provided structured peer scrutiny and category refinement.

Traceability was ensured through four supplementary materials. Supplementary Table S1 provides the source inventory; Supplementary Table S2 summarizes secondary evaluation evidence; Supplementary Table S3 links documentary sources to analytic domains, raw excerpts, and interpretive claims; and Supplementary Table S4 presents the cross-paradigm design-feature comparison underlying the identified design-level tensions and the resulting hybrid architecture.

### Ethics considerations

This study analyzed publicly available and/or published documents and did not involve the recruitment of human participants or the collection of identifiable personal data; therefore, formal research ethics approval was not required.

## Results

Comparative inductive analysis of MRMI and ETS identified four recurrent design-level tensions shaping disaster preparedness outcomes. These tensions emerged through systematic within-case coding of each paradigm’s design features, followed by cross-case comparison and iterative clustering of recurring contrasts. The resulting categories were: (1) divergent disaster conceptualization, (2) fidelity emphasis imbalance, (3) incident-centric temporal framing, and (4) inconsistent translation into organizational learning. Supplementary Table S2 summarizes the secondary evaluation literature used to contextualize the reported strengths and limitations. Supplementary Table S3 links documentary excerpts to analytic domains and interpretive claims. Finally, Supplementary Table S4 presents the cross-paradigm comparison underpinning the identified design-level tensions.

### Divergent disaster conceptualization

Within the MRMI documentation (curricula, manuals, and scenario templates), disasters are consistently framed as governable surge events managed through predefined command structures, standardized triage doctrine, and explicit role alignment [17, 19, 22–26]. Governance architecture and coordinated escalation pathways are foregrounded, and success is implicitly linked to doctrinal adherence and structured coordination across the chain of response.

Within the ETS documentation (manuals, scenario descriptions, and evaluation materials), disasters are instead framed as unstable operational environments characterized by overload, bottlenecks, and performance variability [20, 27–30]. Scenario architecture emphasizes uncertainty, time compression, resource saturation, and communication strain. Rather than centering governance structures, ETS foregrounds the operational consequences of system stress.

When compared across paradigms, this contrast informed the category “divergent disaster conceptualization.” MRMI emphasizes order and governability within surge-oriented architecture, whereas ETS emphasizes instability and adaptive coping under degraded operational conditions. Both paradigms represent authentic disaster properties, but they prioritize different mechanisms of system functioning. Illustrative documentary excerpts supporting this contrast are traceable in Supplementary Tables 2 and 3.

### Fidelity emphasis imbalance

Simulation fidelity is treated here as a multidimensional construct referring to the degree and type of realism represented within a simulation, rather than as a simple high-versus-low property [9, 18]. Within the MRMI materials, fidelity is primarily cognitive and conceptual. The MACSIM system links clinical decision-making to structured triage logic and governance reasoning, reinforcing shared mental models and command clarity [17, 19, 23–26]. Stress exposure is present through surge complexity but is not systematically amplified through inject-driven uncertainty or resource collapse.

Within the ETS materials, fidelity is primarily behavioral and environmental. Real-time patient flow, bottleneck formation, and resource constraints are central design elements [27–30]. Stress-induction mechanisms, including time compression and task saturation, are operationalized within the scenario structure. Governance and ethical escalation pathways are present but are less explicitly modeled.

Cross-case comparison revealed a consistent fidelity imbalance: MRMI strengthens doctrinal coherence but insufficiently tests behavioral breakdown under operational load. In contrast, ETS exposes performance variability but does not consistently anchor adaptative responses within explicit governance and ethical frameworks. This trade-off informed the second design-level tension. Examples of how each paradigm emphasizes different fidelity dimensions are summarized in Supplementary Table 3, with supporting source excerpts provided in Supplementary Table 2.

### Incident-centric temporal framing

The temporal scope was examined through scenario duration, time-handling mechanisms, and representations of system degradation. Within the MRMI scenarios, exercises are largely bounded to initial surge coordination and early patient management phases [19, 22–24]. Within the ETS scenarios, realism is concentrated in the early congestion phase, with limited modeling of sustained attrition or multi-day recovery dynamics [27–30].

Cross-case comparisons showed that both paradigms prioritize the early phases of response. A negative-case review of scenario documentation found no systematic mechanisms for modeling fatigue cycles, supply chain erosion, ethical escalation over time, or risks associated with the recovery phase. In contrast, the disaster evidence literature highlights nonlinear deterioration and sustained operational strain as defining features of real-world events [15]. This recurring contrast informed the third design-level tension: simulations labeled as “disaster” exercises often remain structurally optimized for incident management rather than prolonged system degradation. The limited representation of prolonged degradation across both paradigms is illustrated in Supplementary Table 3 and benchmarked against disaster evidence presented in Supplementary Table 2.

### Inconsistent translation into organizational learning

Debriefing and evaluation mechanisms were examined using a predefined decision rule. Translation mechanisms were coded as present only when documentation specified: (i) structured capture of system-level findings, (ii) assignment of responsibility or ownership, and (iii) explicit linkage to plan revision, SOP modification, or governance processes.

Within the MRMI materials, debriefing commonly focuses on reflection, coordination review, and clinical reasoning [17, 22], but explicit QI handover pathways are specified inconsistently. Within the ETS materials, debriefing emphasizes experiential learning and team reflection [27–30]; however, formal mechanisms for translating findings into structured organizational change are described inconsistently.

Cross-case comparison revealed that while both paradigms include reflective components, neither consistently embeds standardized translation mechanisms within the simulation architecture. Negative-case review did not identify systematic requirements for action assignment, timeline specification, or documented governance feedback loops in either paradigm. This pattern informed the fourth design-level tension: simulation frequently generates meaningful learning experiences but does not reliably translate those insights into structured organizational improvement. Reported debriefing and translation mechanisms, including, including gaps in explicit follow-up structures, are summarized in Supplementary Tables 1–3.

### Synthesis of findings

Taken together, these four design-level tensions characterize the principal structural differences between MRMI and ETS and clarify the simulation design challenges addressed in the following section.

## Discussion

This analysis suggests that disaster preparedness outcomes depend less on the mere presence of simulation than on how simulation is designed [8, 10, 12]. Specifically, preparedness may be shaped by how disasters are conceptualized, which dimensions of realism are emphasized, how temporal dynamics are represented, and whether learning is formally translated into organizational change [5, 9, 18]. The four design-level tensions identified in the Results–divergent disaster conceptualization, fidelity emphasis imbalance, incident-centric temporal framing, and inconsistent translation into organizational learning–help explain why simulation can enhance governance alignment or operational coping while still leaving gaps in preparedness for sustained system degradation.

These findings support reframing disaster simulation not simply as an educational activity but as a potential translational systems intervention. By comparing MRMI and ETS, this analysis suggests that simulation can function as a structured method for examining how emergency care systems function under conditions of surge, uncertainty, and resource constraint, while identifying vulnerabilities in coordination, governance, and resource management. When intentionally designed, simulation may provide a mechanism for converting observed system stress into actionable organizational improvement [11, 44]. This interpretation aligns with socio-technical systems theory and resilience engineering, which emphasize that safety and effectiveness in complex environments emerge from adaptive coordination under variability rather than from adherence to protocols alone [35, 36].

## Interpreting the four findings in relation to disaster preparedness

### 1. Divergent disaster conceptualization

MRMI and ETS embody distinct yet complementary conceptions of disasters. MRMI emphasizes governable surge managed through the predefined command and coordination architecture, reinforcing doctrine, role clarity, and escalation pathways [17, 19, 23]. In contrast, ETS emphasizes instability, overload, and adaptive coping under degraded operational conditions [20, 28]. From a socio-technical systems perspective, these approaches can be understood as representing two complementary dimensions of disaster response: governance and adaptation [35, 36]. However, when used independently, each paradigm risks overemphasizing one dimension at the expense of the other. MRMI may strengthen governance alignment without sufficiently stress-testing system performance, whereas ETS may expose operational fragility without consistently anchoring adaptation within explicit decision authority and ethical frameworks.

### 2. Fidelity emphasis imbalance

The comparison revealed a systematic imbalance in fidelity emphasis between cognitive fidelity and behavioral realism within two broadly intermediate-fidelity simulation paradigms. MRMI strengthens conceptual coherence and governance logic [17, 19, 23], while ETS exposes performance variability, bottlenecks, and communication breakdowns under stress [20, 27]. Effective disaster preparedness requires both dimensions: shared mental models and tested adaptive capacity. Without behavioral stress-testing, doctrine may remain unchallenged; without governance anchoring, stress exposure may produce valuable insights without clear accountability [25–29]. This fidelity imbalance is not a flaw of either paradigm, but a structural limitation when each operates alone.

### 3. Incident-centric temporal framing

Both paradigms privilege the early surge-response phase, reflecting a common incident-centric bias in disaster simulation. However, disasters are defined not only by their initial impact but also by sustained degradation, attrition, ethical escalation, and recovery complexity [5, 9, 15, 18]. When exercises conclude once throughput stabilizes, they fail to adequately test prolonged system strain and the cumulative consequences of decisions made under uncertainty. Resilience engineering highlights the ability to anticipate and monitor over time as critical system capacities [36]. Simulations that do not extend beyond the early phases cannot adequately assess these dimensions [45, 46].

### 4. Inconsistent translation into organizational learning

Finally, although both paradigms include reflective elements, the translation of findings into structured organizational change is embedded inconsistently [7, 32, 33]. As a result, learning frequently remains at the individual or team level rather than being systematically translated into organizational preparedness improvements. From a socio-technical systems perspective, reflection should function not only as a process of cognitive sense-making but also as a mechanism for the joint optimization of social and technical elements [33, 35]. When debriefing processes lack explicit action ownership, implementation timelines, or governance handover mechanisms, simulation risks becoming episodic rather than transformative [47].

### A staged hybrid architecture as a system response

To address these limitations, this study proposes a staged hybrid architecture that integrates governance clarity (MRMI), operational friction (ETS), longitudinal dynamics, and a formal translation mechanism. The goal is not simply to “combine courses,” but to integrate functions that directly address the four identified design-level tensions. From a socio-technical systems perspective, governance structures and operational stressors are not discrete layers but co-evolving elements that interact dynamically under real-world conditions. The staged architecture presented here should therefore be interpreted as an analytic framework and design-oriented proposal rather than as a validated operational sequence. Real disasters involve simultaneous and often conflicting pressures across technical, organizational, social and political domains. From a resilience engineering perspective, the apparent clarity of staged transitions may also obscure latent system vulnerabilities, particularly those that emerge during rapid scaling or prolonged operational stress.

Although the staged model provides a structure for analysis and training, it does not eliminate the inherent non-linearity and unpredictability of disaster systems. These considerations highlight the importance of testing the model under conditions of variability and disruption rather than assuming a stable progression between stages.

#### Stage 1: Governance and doctrine alignment (MRMI-informed)

This stage establishes a common operational language across EDs, EMS, hospital command structures, and supporting agencies. Key elements include escalation logic, ethical principles, and accountability mechanisms. The primary purpose of this stage is to address the divergent disaster conceptualization tension by making the “work-as-imagined” architecture explicit [2, 3, 11, 48].

#### Stage 2: Structured incident response simulation (MRMI core)

This stage involves a time-bounded surge scenario designed to test coordination pathways, triage doctrine, and role interfaces across the chain of response. This objective is to strengthen cognitive fidelity and identify governance bottlenecks under controlled conditions [2, 48, 49].

#### Stage 3: Controlled disruption and operational stress testing (ETS-informed inject layer)

This stage introduces operational friction (capacity constraints, communication overload, shifting priorities, and partial information). Its purpose is to evaluate whether doctrine and coordination processes remain effective under conditions of performance variability. This stage is intended to directly address the fidelity emphasis imbalance by embedding behavioral realism without abandoning system-level reasoning [28, 30, 31].

#### Stage 4: Longitudinal “time-jump” degradation and recovery challenges

This stage advances the scenario into later operational phases to evaluate fatigue, workforce attrition, resource depletion, ethical escalation, and the consequences of earlier decisions. The objective is to address the incident-centric temporal framing tension and align simulation more closely with the sustained operational demands of real-world disasters [7, 32, 33].

#### Stage 5: Structured reflective integration and translation (3LC-style)

**Fig. 3:**
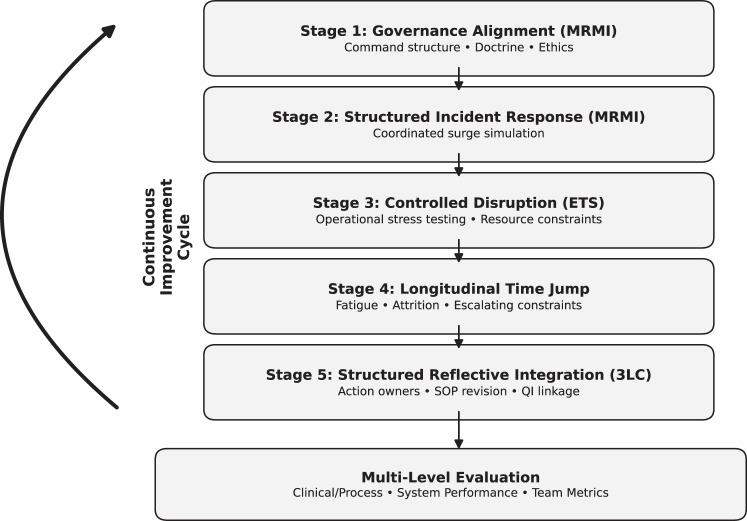
Staged hybrid disaster simulation model as a translational systems intervention. The figure presents a simplified conceptual framework, rather than a complete operational protocol. Five sequential but iterative stages integrate complementary MRMI and ETS functions: (1) governance alignment, including doctrine, command structures, and ethical principles; (2) structured incident response simulation, including coordinated surge management; (3) controlled disruption, including stressors, communication overload, and resource constraints; (4) longitudinal time-jump, including fatigue, workforce attrition, escalating constraints, and recovery-phase challenges; and (5) structured reflective integration, including debrief-to-action processes, Standard Operating procedure (SOP) revision, quality improvement (QI) initiatives, and assigned action ownership. A multi-level evaluation layer spans all stages and links clinical/process indicators, system-performance indicators, and team/coordination indicators. Examples of these evaluation domains, including CSCATTT-based collaborative factors and collaboration-learning-utility (CLU) measures, are provided in appendix A. Abbreviations: MRMI = Medical response to Major Incident; ETS = Emergo Train System; SOP = Standard Operating Procedure; QI = quality Improvement; CSCATTT = communication, situational awareness, cooperation, coordination, accountability, leadership, task management, and team orientation; CLU = collaboration-learning-utility

This stage concludes with an explicit translation workflow that produces organizational outputs rather than reflection alone. Key outputs include: (i) a documented list of observed system vulnerabilities framed as socio-technical mismatches; (ii) designated action owners; (iii) specified revisions to disaster plans/SOPs; and (iv) a follow-up verification plan. The primary purpose of this stage is to address the organizational learning translation tension by explicitly linking simulation findings to quality improvement and governance processes [33, 47]. Together, the five stages form an internally coherent architecture. Governance establishes the organizational framework; structured incident response simulations test execution; operational stress testing probes adaptive capacity; time-jump challenges reveal the effects of system degradation; and structured reflection converts findings into proposed organizational improvements (Fig. 3).

### Integrated evaluation logic

The proposed hybrid architecture is designed to enable multi-level evaluation aligned with the four identified design-level tensions. It combines MRMI-derived clinical and process indicators (e.g., triage accuracy, protocol adherence, outcome-linked decision-making) with ETS-informed system-performance indicators (e.g., throughput, bottlenecks, resource saturation, and coordination delays). In addition, explicit team and coordination metrics–such as CSCATTT-based collaborative factors and Collaboration-Learning-Utility (CLU) measures within 3LC frameworks–allow non-technical aspects of performance to be assessed across all stages. By integrating clinical, system, and team-level domains, the framework is intended to strengthen organizational learning translation and align evaluation processes with WHO EMT governance and quality standards [34]. Appendix A provides an infographic summary of the integrated evaluation logic. CSCATTT-based collaborative factors and CLU measures are presented as illustrative examples of how team coordination, learning value, and practical utility may be incorporated into an evaluation of the staged hybrid framework.

### Relationship to modular and multidisciplinary disaster education (including PRAD-MED)

Modular programs such as PRAD-MED demonstrate the feasibility of staged disaster education that integrates governance-oriented system modeling and structured reflection within time-limited formats while supporting multidisciplinary participation [32]. However, programs that emphasize doctrine, governance, and coordination without explicitly incorporating operational stress testing may not fully represent system vulnerabilities that emerge under conditions of realistic overload [50–53].

The proposed hybrid framework extends modular staging by embedding a dedicated operational stress-testing layer informed by ETS, while preserving governance clarity and structured translation mechanisms that support plan revision and interagency preparedness. This broader approach reinforces that disaster medicine intersects with emergency medicine and public health through shared concerns such as surge sustainability, equity in triage, population risk management, and cross-sector coordination [3, 54–57].

### Implications for emergency medicine, disaster management, and public health practice

For emergency departments and EMS systems, preparedness cannot be inferred from protocol mastery alone. It also requires the capacity to coordinate and adapt under conditions of constraint while maintaining situational awareness and a disaster-oriented mindset [3, 5, 11]. Accordingly, simulations should assess not only whether teams can follow plans, but also whether systems remain functional when those plans are subjected to stress. For hospitals and regional preparedness bodies, the staged model offers a structured mechanism for aligning simulation with governance cycles. In this framing, simulation functions as a periodic systems audit that generates actionable outputs—such as SOP revisions, logistics adjustments, staffing contingencies, and ethical escalation pathways—rather than serving as a standalone training exercise.

These implications inform the staged hybrid architecture proposed here. By integrating governance-oriented modeling, operational stress testing, longitudinal degradation modeling, and structured reflective translation, the framework is positioned as a mechanism for translating observed system vulnerabilities into measurable preparedness and quality improvement actions aligned with WHO EMT benchmarks [2, 34, 48, 49].

### Future research and evaluation priorities

Because this study is design-based and documentary in nature, the staged model should be regarded as a theoretically grounded framework requiring empirical feasibility testing and validation. Priority next steps include: (i) feasibility testing of staged delivery formats; (ii) evaluation of organizational outputs (e.g., documented plan revisions, governance decisions, procurement actions, or staffing changes); (iii) measurement of adaptive coordination under stress (behavioral markers); and (iv) assessment of durability over time through repeated stimulation cycles. Such evaluation would strengthen the evidence base for simulation as a translational preparedness intervention rather than a one-off educational exercise [42, 58–63].

To ensure that the proposed staged hybrid architecture remains empirically testable, it is important to define the conditions under which the model could be refuted. The model would be challenged if implementation of staged simulation fails to produce measurable organizational outputs, such as documented revisions to disaster plans, changes in governance structures, or improvements in coordination performance under stress. Similarly, if staged approaches do not demonstrate improved system-level adaptability compared with single-paradigm exercises, or if the sequential structure fails to capture or enhance performance under prolonged, non-linear disruption, the validity of the model would be weakened. Collectively, these criteria provide a basis for future empirical testing and potential falsification.

### Limitations

This study is a qualitative, design-based comparative analysis of MRMI and ETS using documentary sources and theory-informed interpretation. It does not directly observe course delivery, nor does it quantify behavioral performance, team coordination, or patient outcomes. In addition, simulation quality, fidelity, and modality were inferred from documentary materials rather than directly observed during enacted course delivery, which may limit the precision with which implementation realism and local variation can be characterized. Participant evaluation data cited (e.g., perceived realism, confidence, and self-assessed competence gains) were drawn from published reports rather than collected through a standardized study protocol. As a result, these data cannot be interpreted as causal evidence of effectiveness.

The unit of analysis was simulation design features, which supports analytic transferability but may under-represent the influence of local context on implementation (e.g., instructor expertise, institutional doctrine, resource constraints, and national command structures). Benchmarking against the WHO EMT standards [34] strengthens external grounding for governance and coordination but may not fully capture preparedness priorities in non-EMT or domestic emergency management systems.

Although analytic credibility was strengthened through audit trails, constant comparison, triangulation across documentary types and interpretive lenses (STS/RE), and structured peer review between the two authors, interpretive bias cannot be entirely excluded. As with all design-based research, the proposed hybrid framework represents a theoretically grounded model that requires empirical testing and validation. Future work should evaluate staged hybrid implementation using mixed-methods designs, including behavioral indicators of adaptive coordination, defined organizational outputs (e.g., documented SOP revisions and logistics adjustments), and longitudinal follow-up to assess sustainability and real-world impact.

## Conclusion

Disaster preparedness depends not simply on whether simulation is used, but on how disasters are conceptualized, which dimensions of realism are prioritized, how temporal dynamics are represented, and whether learning is systematically translated into organizational change. This comparative design-based analysis suggests that MRMI and ETS are complementary, yet individually incomplete. MRMI appears to strengthen governance and doctrinal coherence, whereas ETS appears to reveal operational fragility and performance variability under stress. Both paradigms, however, tend to retain incident-centric temporal horizons and to inconsistently operationalize the translation of exercise findings into system-level improvements.

To address these structural limitations, we propose a staged hybrid framework that integrates governance alignment, structured incident simulation, operational stress testing, longitudinal degradation modeling, and structured reflective translation. This framework should be understood as a theoretically grounded, design-oriented proposal rather than an empirically validated intervention. It may support the use of disaster simulation as a translational systems intervention by helping organizations identify socio-technical vulnerabilities and convert simulation findings into accountable governance, operational, and quality improvement actions. Empirical validation is required to assess its feasibility, organizational impact, and outcome relevance across emergency medicine, disaster management, and public health contexts.

## Electronic supplementary material

Below is the link to the electronic supplementary material.

Supplementary Material 1

Supplementary Material 2

## Data Availability

All materials analyzed are published sources cited in the reference list. The analytic extraction matrix and framework iteration memos are available from the corresponding author upon reasonable request.

## Abbreviations

DBR: Design-Based Research
EMT: Emergency Medical Team
ETS: Emergo Train System
MACSIM: Mass Casualty Simulation
MRMI: Medical Response to Major Incidents
QI: Quality Improvement
RE: Resilience Engineering
SOP: Standard Operating Procedures
STS: Socio-Technical System
UNDRR: United Nations Disaster Risk Reduction
WHO: World Health Organization
