# Supplementary figures and images for: Reframing disaster simulation as a translational systems intervention: a design-based comparative analysis of MRMI and the Emergo Train System (ETS)
Source: BMC Emerg Med. 2026 Jul 22;26:202. doi: 10.1186/s12873-026-01699-1 (PMC13401294; doi:10.1186/s12873-026-01699-1)

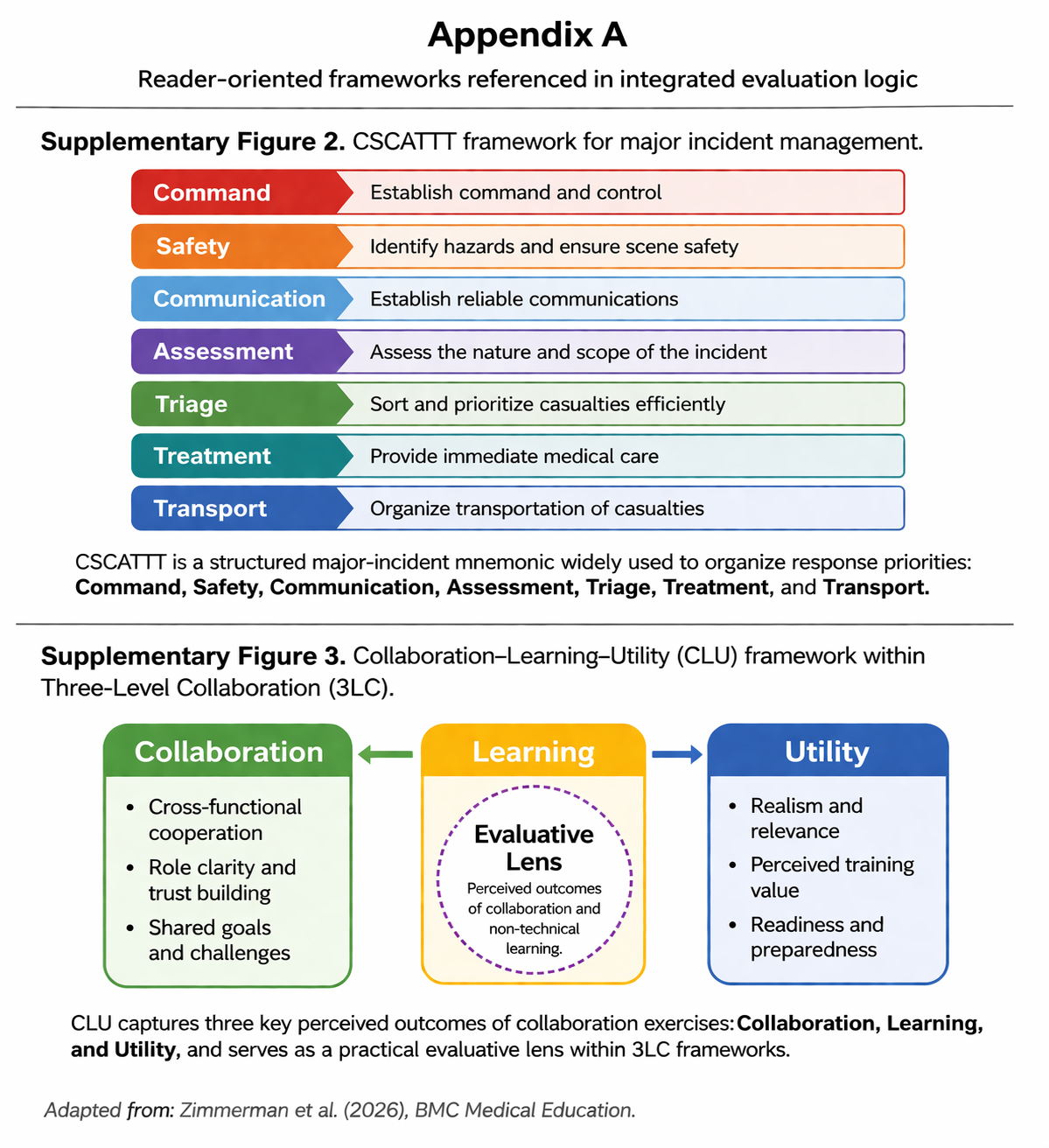

Supplement: Supplementary file 1 — Supplementary Material 1 [file 12873_2026_1699_MOESM1_ESM.png]
