# Supplementary material for: Reframing disaster simulation as a translational systems intervention: a design-based comparative analysis of MRMI and the Emergo Train System (ETS)
Source: BMC Emerg Med. 2026 Jul 22;26:202. doi: 10.1186/s12873-026-01699-1 (PMC13401294; doi:10.1186/s12873-026-01699-1)
Supplement: Supplementary file 2 — Supplementary Material 2 [file 12873_2026_1699_MOESM2_ESM.docx]

**Supplementary table template**

**Supplementary Table S1. Source inventory**

This table was added to improve reproducibility and source transparency. It lists the documentary corpus used in the analysis by source identifier, title or descriptive label, source type, analytic category, access method, access date, access status, and main analytic contribution. Access-controlled or collaborative materials are described at a level that protects proprietary details while indicating their role in the analysis.

| **ID** | **Title/document** | **Source type** | **Category** | **Access method** | **Access date** | **Access status** | **Analytic contribution** |
| --- | --- | --- | --- | --- | --- | --- | --- |
| MRMI-01 | Medical Response to Major Incidents (MRMI) website/material | Official course-provider source | MRMI | Public website | May 25, 2026 | Public | Governance, course structure, scenario architecture |
| MRMI-02 | Assessment of the accuracy of the Medical Response to Major Incidents (MRMI) course for interactive training | Peer-reviewed evaluation article | MRMI | Institutional library/database | May 25, 2026 | Published/public | Patient modeling, course evaluation, process logic |
| MRMI-03 | Education and training for major incidents through the MRMI course | Peer-reviewed article | MRMI | Institutional library/database | May 25, 2026 | Published/public | Course delivery, roles, chain-of-response logic |
| MRMI-04 | Development and evaluation of a new simulation model for interactive training | Peer-reviewed article | MRMI/MACSIM | Institutional library/database | May 25, 2026 | Published/public | MACSIM design, patient cards, interactive training |
| MRMI-05 | Determination of hospital surge capacity for mass-casualty incidents | Peer-reviewed article | MRMI/MACSIM | Institutional library/database | May 25, 2026 | Published/public | Surge capacity, hospital resource modeling |
| MRMI-06 | Mass casualty incident training using MACSIM | Peer-reviewed article | MRMI/MACSIM | Institutional library/database | May 25, 2026 | Published/public | Training design, surge and triage logic |
| MRMI-07 | Assessment of hospital surge capacity using MACSIM | Peer-reviewed article | MRMI/MACSIM | Institutional library/database | May 25, 2026 | Published/public | Hospital capacity, outcome-linked decision logic |
| ETS-01 | Emergo Train System website/material | Official course-provider source | ETS | Public website | May 25, 2026 | Public | ETS methodology, exercise structure |
| ETS-02 | The Emergo Train system for training and testing disaster preparedness | Foundational article | ETS | Institutional library/database | May 25, 2026 | Published/public | ETS origin, training and testing logic |
| ETS-03 | Pandemic preparedness and response testing using the Emergo Train System in a local health district in Sydney, Australia | Evaluation/report article | ETS | Institutional library/database | May 25, 2026 | Published/public | Pandemic application, operational testing |
| ETS-04 | Studying distributed cognition of simulation-based team training | Peer-reviewed article | ETS/team cognition | Institutional library/database | May 25, 2026 | Published/public | Distributed cognition, teamwork, operational realism |
| ETS-05 | Benefits of low-fidelity simulations like Emergo Train System for healthcare providers emergency preparedness | Scoping review | ETS | Institutional library/database | May 25, 2026 | Published/public | Low-fidelity simulation, perceived benefits and limitations |
| ETS-06 | Reflection in teams for training of prehospital command and control teams | Conference paper/report | ETS/command-and-control | Public repository | May 25, 2026 | Public | Reflection, team learning, command-and-control training |
| BEN-01 | WHO simulation exercise manual: practical guide and tool for planning, conducting and evaluating simulation exercises | International manual/standard | Benchmark | Public WHO website | May 25, 2026 | Public | Exercise planning, conduct, evaluation logic |
| BEN-02 | WHO Emergency Medical Teams: minimum technical standards and recommendations for rehabilitation | International standard | Benchmark | Public WHO website | May 25, 2026 | Public | Governance, quality systems, clinical coordination |
| BEN-03 | The ethics of disaster management | Peer-reviewed ethics article | Benchmark/ethics | Institutional library/database | May 25, 2026 | Published/public | Ethical escalation, scarcity, decision-making |
| THE-01 | Systematic review on the current state of disaster preparation simulation exercises | Systematic review | Disaster simulation evidence | Institutional library/database | May 25, 2026 | Published/public | Exercise design, preparedness evidence |
| THE-02 | Simulators and the simulation environment: getting the balance right in simulation-based surgical education | Peer-reviewed article | Simulation theory | Institutional library/database | May 25, 2026 | Published/public | Fidelity concepts and simulation balance |
| THE-03 | Use of simulation in full-scale exercises for response to disasters and mass-casualty incidents | Scoping review | Simulation/disaster evidence | Institutional library/database | May 25, 2026 | Published/public | Full-scale exercises and design features |
| THE-04 | Use of high-fidelity simulation technology in disasters | Integrative review | Simulation/disaster evidence | Institutional library/database | May 25, 2026 | Published/public | High-fidelity simulation and disaster training |
| THE-05 | Examining what features of mass casualty exercise design are important for improving practitioner learning | Report | Exercise design evidence | Public/institutional access | May 25, 2026 | Public/report | Exercise features and practitioner learning |
| THE-06 | Proactive postgraduate education in disaster medicine and preparedness for enhanced disaster management | Peer-reviewed article | PRAD-MED/modular education | Institutional library/database | May 25, 2026 | Published/public | Staged education and reflective components |
| THE-07 | The three-level collaboration exercise: impact of learning and usefulness | Peer-reviewed article | 3LC/evaluation | Institutional library/database | May 25, 2026 | Published/public | Reflection, CLU measures, collaboration learning |
| THE-08 | Value inference in sociotechnical systems | Conference paper | Socio-technical systems | Institutional library/database | May 25, 2026 | Published/public | Socio-technical interpretation |
| THE-09 | Safety-II and resilience engineering in a nutshell | Peer-reviewed article | Resilience engineering | Institutional library/database | May 25, 2026 | Published/public | Resilience engineering concepts |
| THE-10 | Developing a conceptual framework for flexible surge capacity based on complexity and collaborative theoretical frameworks | Peer-reviewed article | Surge capacity/systems | Institutional library/database | May 25, 2026 | Published/public | Flexible surge capacity and coordination |
| THE-11 | Community-based response to the COVID-19 pandemic: case study of a home isolation center using flexible surge capacity | Peer-reviewed article | Surge capacity/systems | Institutional library/database | May 25, 2026 | Published/public | Operational surge and community response |
| THE-12 | Analyzing shared situational awareness in disaster simulations | Peer-reviewed article | Situational awareness | Institutional library/database | May 25, 2026 | Published/public | Situational awareness in exercises |
| THE-13 | Facilitating teamwork in emergency management | Peer-reviewed article | Teamwork/emergency management | Institutional library/database | May 25, 2026 | Published/public | Teamwork and coordination |

MRMI: Medical Response to Major Incidents

ETS: Emergo Train System

BEN: Benchmarked

THE: Theoretical

**Supplementary Table S2: Published Participant Perceptions and Evaluative Feedback on MRMI and ETS (secondary documentary)**

| Domain | MRMI – Reported Participant Perceptions | ETS – Reported Participant Perceptions | Source Type |
| --- | --- | --- | --- |
| Perceived realism | High perceived realism of MACSIM patient cards and structured chain-of-response modelling; majority rated scenario accuracy as good/very good | High perceived operational realism despite low-fidelity tabletop format; realism attributed to time pressure and bottleneck exposure | Published course evaluations; program reports [17,22,27,30] |
| Knowledge/competence gain | Significant self-reported increases in disaster-response knowledge and coordination skills across prehospital, hospital, and administrative roles | Increased confidence in managing major incidents and improved clarity of emergency roles | Published evaluation summaries; scoping review [17,30] |
| Governance & coordination clarity | Improved understanding of command structure, escalation logic, and inter-unit coordination; strengthened “whole chain of response” awareness | Improved interagency communication and role awareness under stress | Course evaluations; training reports [17,22,27] |
| Stress exposure | Moderate stress exposure; emphasis on structured coordination rather than overload-induced breakdown | Strong exposure to time pressure, congestion, and operational friction; participants report realistic workload strain | Program descriptions; evaluation literature [27–30] |
| Teamwork & collaboration | Strengthened shared mental models and cross-disciplinary coordination | Improved teamwork awareness and adaptive communication under constraint | Published evaluations; scoping review [17,30] |
| Translation into practice | Recognized as practical doctrine suitable for national implementation; structured but variable evidence of formal QI handover | Debrief valued for identifying plan gaps; participants request more frequent exercises; formal QI linkage variably described | Evaluation summaries; training reports [17,27,30] |
| Implementation feedback | High demand in several regions; adaptable to multi-agency context | Requests for digitalization (e.g., DigEmergo); additional instructor guidance recommended | Published program reports [27,30] |
| Scope beyond disaster | Primarily focused on mass-casualty and major incident doctrine | Applied also to surge testing, hospital relocation exercises, and pandemic preparedness contexts | ETS literature; case reports [27,28] |

**Supplementary Table S3: Traceability matrix linking sources to findings**

| Finding/Design-level tension | Claim component | Evidence type | Primary source(s) (doc names/IDs) | Excerpt/concrete example (short) | Extracted feature domain(s) | Analytic step (within-case/cross-case) | Triangulation check (STS/RE/WHO EMT) | Interpretation note/decision rule |
| --- | --- | --- | --- | --- | --- | --- | --- | --- |
| 1. Divergent disaster conceptualization | MRMI frames disasters as governable surge within defined architecture | Curriculum/manual | MRMI manual och scenario template; MACSIM guide | e.g., emphasis on role/structure emphasis, chain-of-response module | Governance; Scenario architecture; Evaluation | Within MRMI feature extraction + coding → Cross-case comparison (MRMI vs ETS) → Negative-case scan (look for design elements emphasizing uncertainty or instability) | WHO EMT: command/coordination alignment; STS: governance logic | Counted as “normative framing” if doctrine specifies predefined roles, escalation pathways, and system architecture as primary control mechanisms |
| 1. Divergent disaster conceptualization | ETS frames disasters as unstable, uncertainty-heavy operational environment | Curriculum/manual + evaluation | ETS instructor guide; ETS scenario notes; published ETS evaluation | e.g., time pressure, bottleneck management, overload injects | Stressors; Resource modeling; Time | Within ETS feature extraction + coding → Cross-case comparison (MRMI vs ETS) → Negative-case scan (look for explicit doctrine or governance scaffolding) | RE: performance variability; STS: breakdowns at interfaces | Counted as “instability framing” if scenario design intentionally amplifies uncertainty/overload (injects, information distortion, time compression) as the primary learning mechanism |
| 2. Fidelity trade-off | MRMI high cognitive fidelity, lower behavioral stress exposure | Curriculum/manual + participant feedback | MRMI manuals; published MRMI eval. summaries | e.g., emphasis on doctrine, triage logic; limited stress induction detail | Patient modeling; Governance; Stressors | Within MRMI coding (cognitive vs behavioral fidelity markers) → Cross-case comparison → Negative-case scan (search MRMI materials for explicit stressor operationalization) | RE: adaptive capacity not fully tested; STS: cognition vs operational coupling | “Behavioral fidelity” coded only when stressors are operationalized (e.g., time distortions, injects, information loss, workload saturation) rather than described as intentions |
| 2. Fidelity trade-off | ETS high behavioral realism, governance often implicit | Curriculum/manual + evaluation | ETS manual; ETS AARs; published ETS evaluations | e.g., role clarity emerges, but governance/ethics not explicit | Stressors; Governance | Within ETS coding (behavioral vs cognitive or system-level markers) → Cross-case comparison → Negative-case scan (search ETS docs for explicit governance or ethics modelling and evaluation) | WHO EMT: governance/clinical governance explicitness; STS: accountability structures | “Implicit governance” coded when accountability, decision authority, escalation, or policy constraints are not explicitly modelled and/or not evaluated (left to emerge informally) |
| 3. Incident-centric temporal scope | Both paradigms emphasize early-phase response; limited prolonged ops | Scenario templates + disaster evidence | MRMI scenarios; ETS scenarios; AARs showing prolonged attrition | e.g., scenarios end at transport completion; contrast with AAR fatigue/attrition | Temporal scope; Evaluation | Within MRMI temporal coding → Within ETS temporal coding → Cross-case comparison → Benchmark check against disaster evidence for prolonged degradation | RE: anticipation/monitoring over time; WHO EMT: sustained capability; STS: drift over time | “Longitudinal gap” asserted only when no explicit mechanism models fatigue/attrition, resource depletion, secondary events, ethical escalation, or recovery transitions (e.g., time-jumps, multi-day phases, cumulative consequences) |
| 4. Translation gap | Debriefing not consistently linked to SOP/QI governance | Manuals + published course descriptions + secondary evaluation literature | MRMI/ETS debrief notes; course descriptions; 3LC literature | e.g., debrief focuses on learning, not action assignment | Debrief/translation; Evaluation; Governance | Within MRMI coding (debrief purpose + outputs) → Within ETS coding → Cross-case comparison (presence/absence of explicit QI handover) → Negative-case scan (search for action logs, named owners, deadlines, SOP triggers) | STS: joint optimization (social + technical change); RE: learning loops/feedback; WHO EMT: quality systems, governance, improvement cycles | Coded as “translation present” only if the debrief specifies system-level outputs (e.g., assigned actions, responsible owner, timeline, SOP revision trigger, escalation to governance/QI committee). If debrief remains reflective without defined organizational handover, coded as “translation gap.” |

**Supplementary Table S4: Structured Design-Feature Matrix (MRMI vs. ETS vs. Hybrid Implication)**

| Design Domain | MRMI | ETS | Identified Tension | Hybrid Design Implication |
| --- | --- | --- | --- | --- |
| Disaster conceptualization | Disaster framed as governable surge within predefined architecture; emphasis on command logic and coordination doctrine | Disaster framed as unstable, overloaded socio-technical environment; emphasis on breakdown and friction | Governance clarity vs. operational instability | Stage 1–2 establish governance architecture; Stage 3 stress-tests instability within that architecture |
| Governance & command structure | Explicit roles, escalation pathways, chain-of-response modelling | Roles operational but governance/ethics often implicit or emergent | Normative structure vs. adaptive improvisation | Explicit governance modelling followed by injects that test escalation and decision authority under stress |
| Scenario architecture | Modular scene → transport → hospital chain; structured decision nodes | Tabletop system modelling units; dynamic flow and bottlenecks | Structured doctrine vs. flow-based realism | Combine modular chain-of-response with dynamic inject-based disruption |
| Patient modeling | MACSIM cards with algorithmic logic; clinical status linked to interventions | Large patient bank; dynamic but flow-oriented; emphasis on movement and prioritization | Clinical outcome logic vs. operational throughput logic | Integrate clinical outcome modelling with operational congestion effects |
| Resource modeling | Surge capacity structured within doctrine; predefined allocation logic | Resource scarcity and bottlenecks emerge dynamically under load | Predefined capacity vs. emergent saturation | Explicit surge modelling + simulated resource depletion and attrition over time |
| Stress induction mechanisms | Limited explicit stress injects; pressure primarily from volume | Time compression, overload, information gaps, coordination breakdown as core design features | Cognitive fidelity vs. behavioral realism | Add structured ETS-style inject layer after governance alignment |
| Temporal scope | Primarily early surge phase; limited modelling of prolonged degradation | Primarily early-phase congestion; limited modelling of sustained fatigue/recovery | Incident-centric bias | Introduce longitudinal time-jump (fatigue, attrition, ethical escalation, recovery risks) |
| Evaluation logic | Success measured by doctrinal adherence and clinical outcome proxies (e.g., avoidable mortality logic) | Success measured by throughput, plan adherence, coordination effectiveness | Clinical/process endpoints vs. system-performance indicators | Multi-level evaluation: clinical + system + team/coordination metrics |
| Team & coordination metrics | Emphasis on role clarity and shared mental models | Emphasis on teamwork under stress and operational communication | Cognitive alignment vs. behavioral coping | Add explicit coordination metrics (e.g., CSCATTT factors; CLU) across stages |
| Debrief structure | Reflective debrief common; action pathway variably specified | Reflective debrief common; QI linkage variable | Reflection present but translation inconsistent | Structured Reflective Integration (3LC) (explicit action owner, timeline, SOP trigger, governance handover) |
| Organizational learning pathway | Learning primarily individual/team-level | Learning primarily experiential/team-level | Limited formal QI translation | Formalized QI capture: vulnerability list → assigned responsibility → follow-up cycle |
| Alignment with WHO EMT standards | Strong in governance, coordination, triage doctrine | Strong in operational realism but governance alignment less explicit | Complementary strengths | Hybrid explicitly benchmarked against EMT coordination, governance, and sustained capability standards |
